# Development of IGF Signaling Antibody Arrays for the Identification of Hepatocellular Carcinoma Biomarkers

**DOI:** 10.1371/journal.pone.0046851

**Published:** 2012-10-11

**Authors:** Qi Zhou, Ying-Qing Mao, Wei-Dong Jiang, Yun-Ru Chen, Ren-Yu Huang, Xiang-Bing Zhou, Ya-Feng Wang, Zhi Shi, Zhong-Sheng Wang, Ruo-Pan Huang

**Affiliations:** 1 Department of Hepatobiliary Surgery, the First Affiliated Hospital, Sun Yat-Sen University, Guangzhou, China; 2 RayBiotech, Inc., Guangzhou, China; 3 RayBiotech, Inc., Norcross, Georgia, United States of America; 4 South China Biochip Research Center, Guangzhou, China; Baylor College of Medicine, United States of America

## Abstract

**Purpose:**

Our objective was to develop a system to simultaneously and quantitatively measure the expression levels of the insulin-like growth factor (IGF) family proteins in numerous samples and to apply this approach to profile the IGF family proteins levels in cancer and adjacent tissues from patients with hepatocellular carcinoma (HCC).

**Experimental Design:**

Antibodies against ten IGF family proteins (IGF-1, IGF-1R, IGF-2, IGF-2R, IGFBP-1, IGFBP-2, IGFBP-3, IGFBP-4, IGFBP-6, and Insulin) were immobilized on the surface of a glass slide in an array format to create an IGF signaling antibody array. Tissue lysates prepared from patient's liver cancer tissues and adjacent tissues were then applied to the arrays. The proteins captured by antibodies on the arrays were then incubated with a cocktail of biotinylated detection antibodies and visualized with a fluorescence detection system. By comparison with standard protein amount, the exact protein concentrations in the samples can be determined. The expression levels of the ten IGF family proteins in 25 pairs of HCC and adjacent tissues were quantitatively measured using this novel antibody array technology. The differential expression levels between cancer tissues and adjacent tissues were statistically analyzed.

**Results:**

A novel IGF signaling antibody array was developed which allows the researcher to simultaneously detect ten proteins involved in IGF signal pathway with high sensitivity and specificity. Using this approach, we found that the levels of IGF-2R and IGFBP-2 in HCC tissues were higher than those in adjacent tissues.

**Conclusion:**

Our IGF signaling antibody array which can detect the expression of ten IGF family members with high sensitivity and specificity will undoubtedly prove a powerful tool for drug and biomarker discovery.

## Introduction

The IGF signaling system plays an important physiological role in regulating cellular proliferation, differentiation, and apoptosis by interacting with specific receptors localized on the cell membrane [1]. The IGF system is composed of ligands (IGF-1, IGF-2, and insulin), receptors (IGF-1R, IGF-2R, insulin receptor (IR), IGF-1R/IR hybrid receptor (HR)), and six high-affinity binding proteins (IGFBP1-6) [2]. IGF-1 and IGF-2 are critical players in fetal development and postnatal life through endocrine, paracrine and autocrine mechanisms [3]. The mitogenic, differentiating and antiapoptotic properties of IGFs are mediated primarily by IGF-1R. Upon binding to IGF-1 or IGF-2, IGF-1R may promote cellular proliferation or inhibit apoptosis through the MEK/ERK or PI3K/Akt signaling pathways, respectively, thereby increasing the risk of carcinogenesis [4]. Among the IGF family proteins, IGF-1, IGF-1R and IGF-2 are positively correlated to cancer formation [5], [6]. In contrast, the IGFBPs are important modulators of metabolism through the high affinity binding of IGFs, which depresses their activity [2], [7]. On the cellular surface, IGFBPs competitively bind IGFs to block their interaction with IGF-1R [8].

The IGF system has drawn much attention in the last decade in both academic field and pharmaceutical companies. Dysregulation of the IGF system has been recognized as a key contributor to a variety of diseases including diabetic diseases, cardiovascular disease, and multiple cancers [9]. Since elevated expression of IGF-1R increases the risk of breast, colon, prostate, and lung cancer, and blocking IGF-1R decreases cell growth and tumor formation, IGF-1R is increasingly recognized by the medical community as a relevant target for investigation in cancer research [10]. More than 30 anticancer drugs targeting IGF-1R, including monoclonal antibodies (mAbs) and tyrosine kinase inhibitors (TKIs), are under evaluation as single agents or in combination therapies [11]. Though inhibiting IGF-1R functions have shown very encouraging results in preclinical conditions, it has been challenging to translate the results from in vitro and animal studies into therapeutic efficacy [12]. Results from clinical study call attention to the complexity of the IGF system. One of the main complexities arises from the fact that the ligands can not only bind with high affinity to their own receptors (e.g., IGF-1→IGF-1R), they can also crosstalk with other receptors with different affinities (e.g. IGF-2→IGF-1R, IR, HR). The serum IGFs level is regulated by those higher affinity IGFBPs. The relative affinities of IGF-1 and IGF-2 vary for the different IGFBPs with IGFBP-1,3,4 having higher affinities for IGF-1 compared to IGF-2 and vice versa for IGFBP-2,5,6. Meanwhile, in addition to their IGF binding functionality, these IGFBPs also possess other IGF-independent functions [13]. In order to have a full picture of the drug efficacy, future anticancer drug development targeting the IGF system is highly recommended to have strategies considering the IGF system in all its complexity.

Gaining insights into the complexity of IGF signaling pathway requires detection of multiple IGF family proteins simultaneously. The current approaches of Western blotting or ELISA to detect individual protein expression levels greatly limits the advancement of IGF research. Antibody arrays have emerged as a novel and necessary technology for simultaneously protein expression profiling and biomarker discovery [14], [15], [16,]. Over the years, our array development efforts have mainly focused on sandwich-based and biotin label-based platforms. Sandwich-based arrays use the same two-antibody detection method as a standard ELISA, allowing high detection sensitivity, specificity and reproducibility [17]. In this report, we describe the development of this new approach to simultaneously detect 10 members of IGF signal family with high specificity and sensitivity. With these IGF antibody arrays, we measured the expression levels of 10 members of IGF signal family and found that IGF-2R and IGFBP-2 were increased in hepatocellular carcinoma (HCC) tissues compared with adjacent tissues.

## Results

### Development of IGF signaling antibody arrays

**Figure 1:**
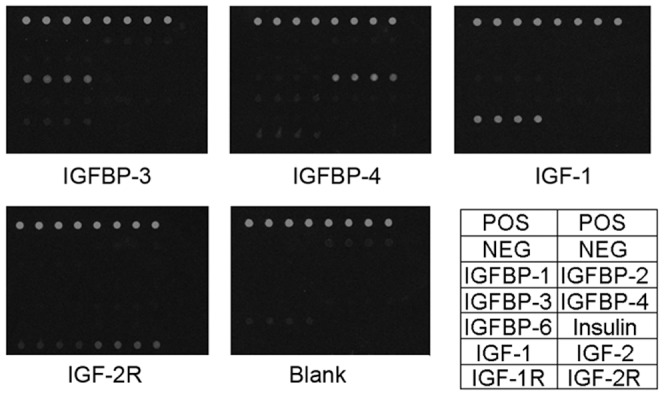
The specificity of IGF signaling antibody array. After incubated with fluorescence conjugated streptavidin, the array was scanned by Genpix 4000B and the specific signal of each subarray was visualized. The signal intensity was extracted by Genpix software, and showed the array specificity as well.

To develop IGF signaling antibody arrays, we first screened commercially available antibodies for suitable antibody pairs. The pairs of antibodies were then used to create an array for simultaneous detection of 10 proteins of IGF signaling family. The overall sensitivity of the array is shown in Table 1; the detected levels of most proteins were at pg/ml to ng/ml range and the minimal detected level was 7.8 pg/ml. The difference in detection sensitivities for individual proteins may be attributable to differences in binding affinity for each antigen-antibody interaction, as well as the binding characteristics of the specific antibody to the solid support. Next, the specificity of antigen-antibody pair was tested. The array was incubated with antigen mixture at a final concentration of 10 ng/ml for each antigen, then detected with individual detection antibodies (Figure 1). The strongest signals between each capture antibody and its corresponding detection antibody are underlined, (Table 2), suggesting a high specificity interaction. Thirdly, the variability was determined by comparing the signals from 4 replicated spots in the same array, 3 different arrays within the same slide and 3 separate arrays from 3 slides. The coefficient of variation (Table 3), suggested that the reliability of the system was good.

**Table 1:** The sensitivity of IGF signaling antibody arrays.

Protein	Sensitivity (pg/ml)	Protein	Sensitivity (pg/ml)
IGFBP-1	7.8	IGF-1	266
IGFBP-2	146	IGF-2	43
IGFBP-3	1756	IGF-1R	92
IGFBP-4	1344	IGF-2R	52
IGFBP-6	84	Insulin	1314

**Table 2:** The specificity of IGF signaling antibody arrays.

	IGFBP-1	IGFBP-2	IGFBP-3	IGFB-4	IGFBP-6	IGF-1	IGF-2	IGF-1R	IGF-2R	Insulin
IGFBP-1	12407	11	37	25	4	11	11	5	72	9
IGFBP-2	18	1550	5	6	4	2	7	6	36	4
IGFBP-3	6	3	1245	54	4	56	2	3	36	3
IGFBP-4	9	4	11	2376	3	8	14	12	44	3
IGFBP-6	18	63	33	38	1957	22	61	20	107	15
IGF-1	1	1	117	12	1	2684	3	1	55	2
IGF-2	1	1	3	3	2	1	12702	6	32	6
IGF-1R	1	2	5	173	1	1	3	709	*1269*	4
IGF-2R	1	1	8	9	1	1	1	19	3261	1
Insulin	5	3	57	10	11	6	16	4	69	2431

**Table 3:** The variability of IGF signaling antibody arrays.

	spot to spot (n = 4)	well to well (n = 3)	slide to slide (n = 3)
CV %	7.68	12.49	14.83

**Figure 2:**
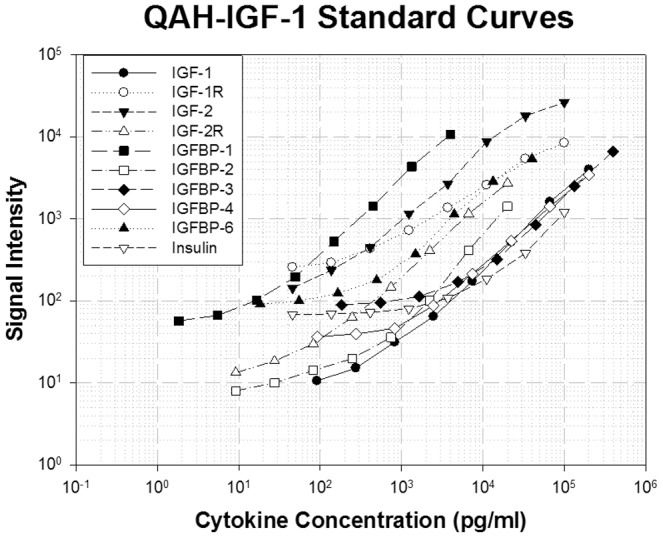
The standard curves of IGF signaling antibody array. Each standard antigen at individual concentration was diluted 3-fold and incubated with array slide. The data was analyzed and the standard curves were established using specific IGF-1R analysis software (R^2^>0.97).

In order to obtain standard curves for each target protein, a mixture of purified antigens was incubated at gradient concentrations on different arrays of the same chip. The concentration of each antigen in the standard mixture was optimized to the sensitivity of the corresponding capture antibody on the chip. The standard curves were expressed as signal intensity versus concentration of each target protein (Figure 2).

### Validation of IGF signaling antibody array

In addition to specificity, sensitivity and variability, we established the stability and accuracy of the antibody array. First, we tested the array with conditioned medium and human serum. We performed a spike-in study by adding recombinant IGF-IR family proteins at various concentrations into the condition media and human sera. The recovery rates of most of the spiked proteins reached up to 80% of the theoretical value (Table 4). The low recovery of few markers may be the result of matrix effects or of the high abundance of particular target protein in the sample.

**Table 4:** The spiking recovery rate for human serum and conditioned media (CM) of IGF signaling antibody arrays.

(pg/ml)	Spiking	CM	CM+Ag	CM%	Serum	Serum+Ag	Serum%
IGFBP-1	2,000	18	1,620	80%	1,752	2,802	52%
IGFBP-2	20,000	33,028	50,505	87%	32,403	52,537	101%
IGFBP-3	150,000	198,02	348,612	100%	28,742	95,730	45%
IGFBP-4	100,000	4,243	120,441	116%	13,480	118,238	105%
IGFBP-6	10,000	11,584	23,051	115%	25,190	34,824	96%
IGF-1	100,000	0	125,179	125%	456	65,565	65%
IGF-2	40,000	0	37,735	94%	0	23,697	59%
IGF-1R	40,000	0	32,480	81%	0	10,300	26%
IGF-2R	10,000	0	7,580	76%	101	2,015	19%
Insulin	40,000	147,829	179,138	78%	3,634	30,801	68%

CM, conditioned media.

### Identification of HCC biomarkers using IGF signaling antibody array

**Figure 3:**
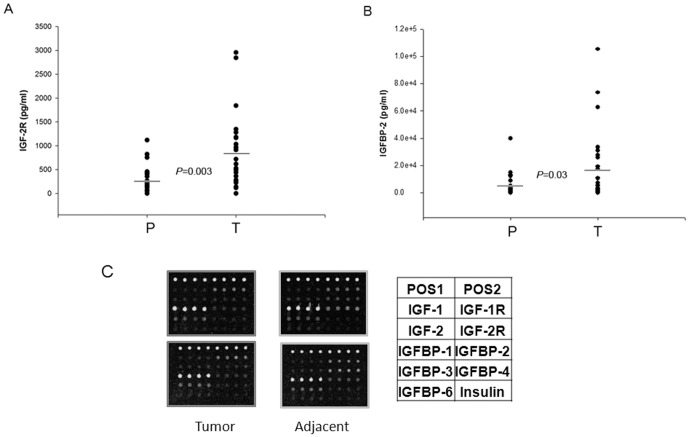
The differential expression of IGF-2R and IGFBP-2. IGF-2R (A) and IGFBP-2 (B) were differentially expressed between tumor (T) and matching paratumorous (P) samples. Liver cancer tissue and adjacent tissue lysates from 25 patients was prepared and incubated with IGF signaling antibody arrays, and the data was statistically analyzed. The average concentrations of IGF-2R and IGFBP-2 expressed differentially from liver cancer and adjacent tissues were compared and the *P*<0.05 (T test). The representative data of Antibody Arrays are shown in (C).

**Figure 4:**
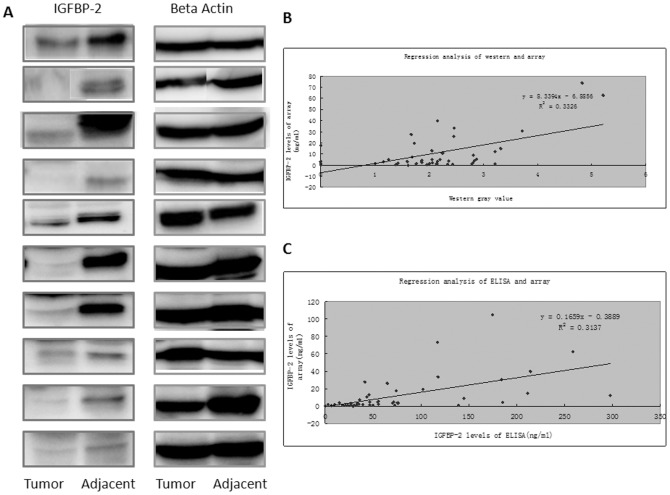
The confirmation of IGFBP-2 expression by Western blotting and ELISA. The expression of IGFBP-2 was further determined by Western blotting analysis and the representative data were shown in (A). The correlation between antibody arrays and Western blotting was determined using regression analysis .and shown in (B). Similarly, the correction between antibody arrays and ELISA was shown in (C).

To exploit the potential application of IGF signaling pathway antibody arrays, we analyzed the expression levels of 10 proteins from IGF signaling pathway in tumor samples and matching paratumorous tissue from 25 patients. Protein levels higher than background plus 2×SD were subjected to adjusted students t test analysis. Two out of 10 proteins (IGF-2R and IGFBP-2) were differentially expressed between tumor samples and matching paratumorous samples, with *P*-values of less than 0.05 (Figure 3). The results were further confirmed by Western blotting analysis. As shown in Figure 4 A, the expression of IGFBP-2 was in general higher in tumor tissue compared with matching paratumorous tissue. Analysis indicates that the expression levels of IGFBP-2 determined by antibody arrays show nice correlation with those determined by either Western blotting or ELISA. Those data further support the reliability of the antibody arrays we have developed.

**Figure 5:**
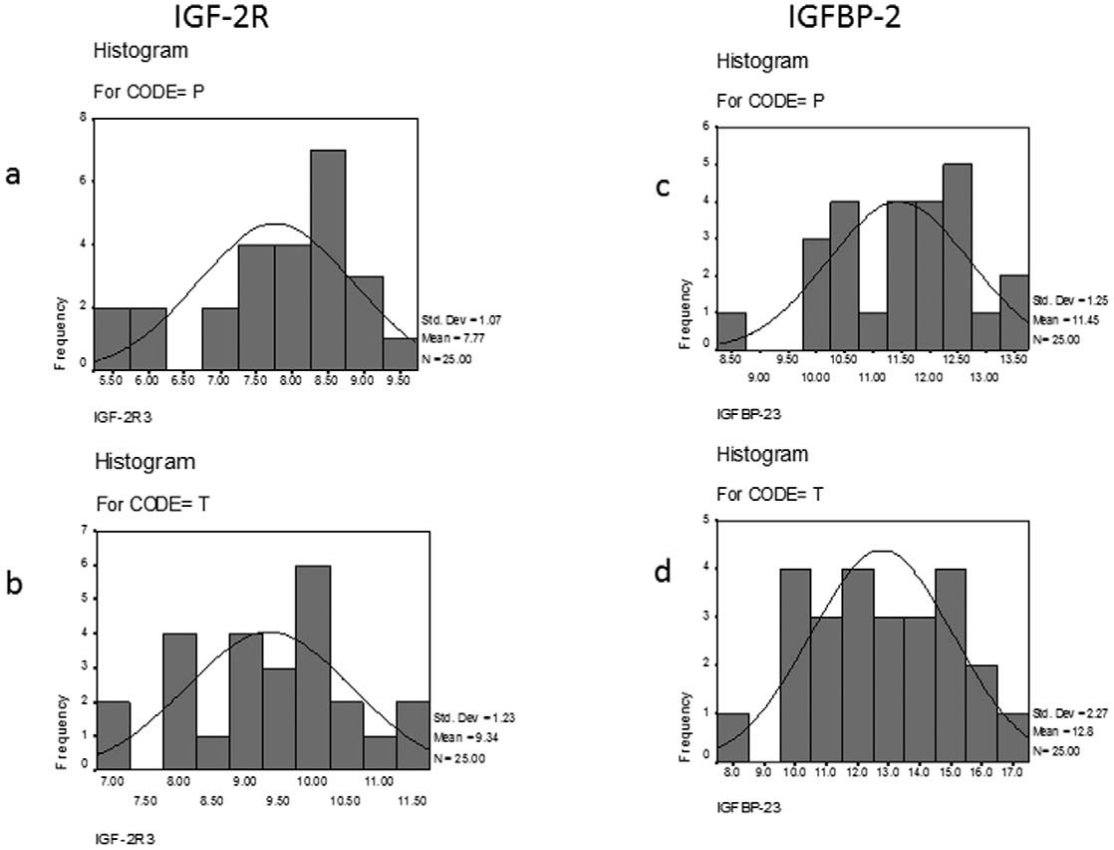
The IGF-2R and IGFBP-2 sample distributions. The normal distributions of IGF-2R (A) and IGFBP-2 (B) were present in both tumor (T) and matching paratumorous (P) samples. Liver cancer tissue and adjacent tissue lysates from 25 patients was prepared and incubated with IGF signaling antibody arrays, and the data was statistically analyzed.

To analyze whether the samples were within normal distribution, both Kolmogorov-Sminov analysis and Shapiro-Wilk analysis were performed on the tumor and paratumorous groups. Figure 5 shows that for both IGF-2R and IGFBP-2, normal distributions were present in both tumor and paratumorous groups, as indicated by *P*>0.05 (Table 5).

**Table 5:** The Tests of Normality^b^ of IGFBP-2 and IGF-2R in 25 pairs of tumor (T) and paratumorous (P) tissues.

		Kolmogorov-Smirnov^a^			Shapiro-Wilk		
	CODE	Statistic	df	Sig	Statistic	df	Sig
IGFBP-2	P	0.135	25	0.200*	0.963	25	0.479
	T	0.117	25	0.200*	0.972	25	0.691
IGF-2R	P	0.127	25	0.200*	0.929	25	0.083
	T	0.11	25	0.200*	0.976	25	0.801

* . This is a lower bound of the true significance.

a . Lilliefors Significance Correction.

b . There are no valid cases for IGFBP-2 and IGF-2R. Statistics cannot be computed.

**Figure 6:**
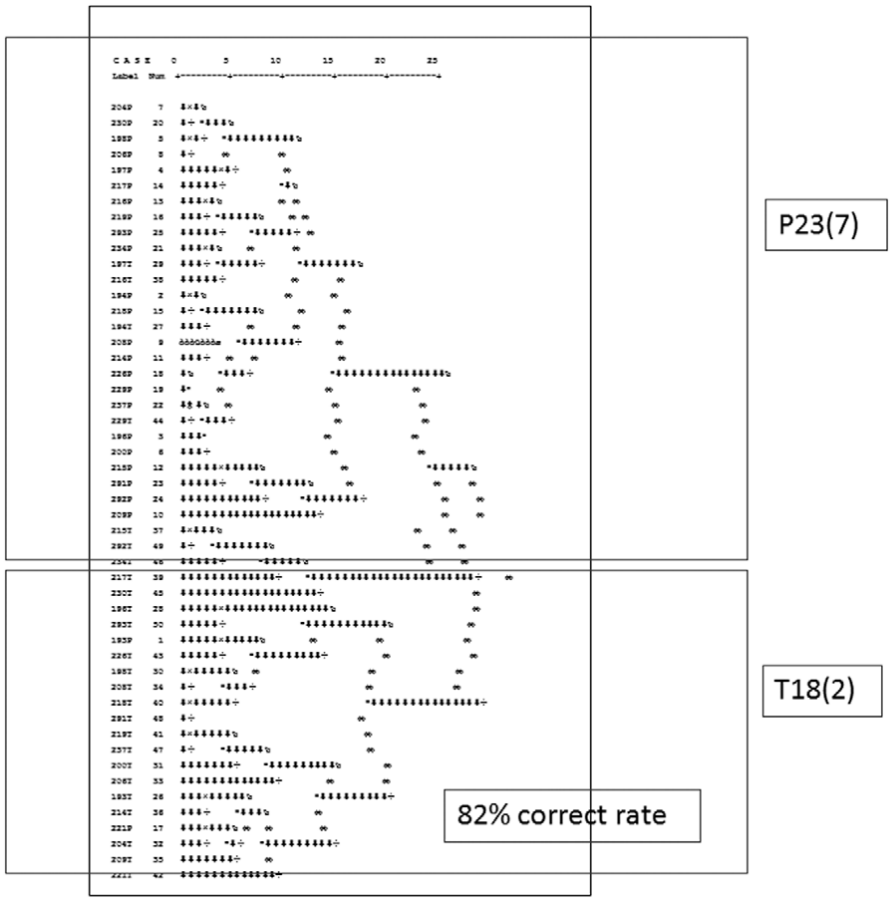
Hierarchical cluster data analysis. Hierarchical cluster analysis results were present in tumor (T) and matching paratumorous (P) samples. Liver cancer tissue and adjacent tissue lysates from 25 patients was prepared and incubated with IGF signaling antibody arrays, and the data was statistically analyzed.

IGF-2R and IGFBP-2 were then used to differentiate between tumor samples and matching paratumorous samples using unsupervised hierarchical cluster analysis. Hierarchical cluster analysis was used in this study to cluster analysis of tumor samples and matching paratumorous tissue samples, in which the object is to group together objects or records that are “close” to one another. A key component of the analysis is repeated calculation of distance measures between objects, and between clusters once objects begin to be grouped into clusters. The outcome is represented graphically as a dendrogram. The initial data for the hierarchical cluster analysis of N objects is a set of object-to-object distances and a linkage function for computation of the cluster-to-cluster distances. Agglomerative methods for hierarchical cluster analysis are of wider use here. In each step, the pair of clusters with smallest cluster-to-cluster distance is fused into a single cluster. As shown in Figure 6, the model used all observations in tumor samples and matching paratumorous samples to fit the model. Overall, 82% (41 out of 50) of individuals were correctly classified when using these two markers to differentiate, including 92% of paratumorous samples (23 out of 25) and 72% of tumor samples (18 out of 25).

## Discussion

The traditional methods for cytokine and signaling protein detection and quantification include ELISA and Western blotting [18]. In these methods, target protein is first immobilized to a solid support. The immobilized protein is then complexed with an antibody that is linked to an enzyme. Detection of the enzyme-complex can then be visualized through the use of a substrate that produces a detectable signal [19]. While these methods work well for a single target protein, the overall procedure is time consuming and requires a large sample volume. Thus, conservation of small, precious sample quantities becomes a risky task. To overcome these issues, we developed a quantitative antibody array using multiplexed sandwich ELISA-based technology which enables accurate determination of the concentration of multiple proteins simultaneously. This system combines the advantages of high sensitivity and specificity of ELISA with the high throughput of the microarray. Like a traditional sandwich-based ELISA, it uses a matched pair of protein specific antibodies for detection [20]. A capture antibody is first bound to the glass surface. After incubation with the sample, the target protein is trapped on the solid surface. A second biotin-labeled antibody is then added, which recognizes a different epitope of the target protein. The protein-antibody-biotin complex can then be visualized through the addition of the streptavidin-labeled Cy3 equivalent dye using a laser scanner. By arraying multiple protein-specific capture antibodies onto a glass support, multiplex detection of proteins in one experiment is made possible. This is not only one of the most efficient methods for protein quantification, but makes it more affordable for quantification of large number of proteins.

HCC is a highly aggressive neoplasm which represents the sixth most common cancer and the third most common cause of death from cancer worldwide [21]. Mainly, HCC results from alcoholic liver disease, metabolic disorders, metastasis of cancer to other parts of the body such as the colon, and in particular, hepatitis C virus (HCV) and hepatitis B virus (HBV) infection [22]. The malignant transformation process of HCC is mediated by a number of factors, particularly the IGF axis with its component ligands, receptors, substrates, and ligand binding proteins [23]. In human HCC tissues, IGF-1 mRNAs were expressed at lower levels than the surrounding normal liver tissues [24]. IGF-2 has been reported to be overexpressed in animal models of hepatocarcinogenesis and in human HCC [25], [26], [27], [28]. In a study where 10 HCC cell lines (including PLC HCC cell line) were tested, all showed elevated IGF-1R mRNA [25]. The levels of IGF-2R protein in human HCC tissues were reduced compared to those in adjacent normal liver tissues [29]. The expression of IGF-2R was significantly lower in several HCC cell lines in vitro, in HCC animal models and in human HCC tissues [30]. In a study comparing IGFBP-1, 3 and 4 levels in human normal liver, cirrhotic liver and HCC, the expression of all three IGFBP-3 mRNA levels was significantly reduced in HCC [31]. The basal serum levels of IGFBP-2 were markedly elevated in HCC [32]. However, due to the lack of available tools, no study thus far has simultaneously examined 10 IGF family proteins. To investigate the potential role of the IGF signaling pathway in liver cancer, we determined the expression levels of 10 IGF family proteins (IGF-1, IGF-1R, IGF-2, IGF-2R, IGFBP-1, IGFBP-2, IGFBP-3, IGFBP-4, IGFBP-6, and Insulin) in 25 pairs of HCC and adjacent tissues using an IGF-1R antibody array. We showed that two proteins, IGF-2R and IGFBP-2, were elevated in HCC compared to adjacent tissues, while the other 8 were not different. The discrepancy between our data and those reported in the literature are probably due to the variance in samples, different modifications of protein and overall profile of IGF family protein, etc. The two proteins we found highly expressed in HCC, IGF-2R and IGFBP-2, both have important roles in tumorigenesis. IGF-2R, also mannose-6-phosphate receptor, is considered a tumor suppressor because it clears IGF-2 from the cell surface to attenuate signaling and loss of function mutations in IGF-2R has been identified in human cancers [33]. Downregulation of the IGF-2R promotes the growth of transformed cells by sustaining IGF-2, which binds to and activates IGF-1R and insulin receptor to increase intracellular growth signals [34]. IGFBP-2 has been found to be a growth factor-promoting tumor, and elevated in the serum of patients with various malignancies [35], [36]. Several IGFBP-2 *in vitro* and *in vivo* models suggested that binding of IGFs by IGFBP-2 has growth-inhibitory consequences, and other functions of IGFBP-2 such as stimulating cell proliferation in an IGF-independent manner must be taken into account when the correlation between IGFBP-2 and tumor growth is examined [37]. The complex pictures of these finding further stress the importance of simultaneous profiling of all IGF family protein. Therefore, further study may help us to elucidate the correlation among IGF family protein and provide insight on the hepatocarcinogenesis and identify new biomarker for hepatocellular carcinoma.

In summary, we have developed an IGF signaling antibody array for simultaneous detection of 10 IGF-1R family proteins. Using this approach, for the first time we were able to simultaneously detect the 10 IGF members from HCC samples and matching paratumorous samples. -We found that the levels of IGF-2R and IGFBP-2 in HCC tissues were higher than those in adjacent tissues, and propose that these two proteins might be new biomarkers of HCC. Our IGF signaling antibody array, which simultaneously and quantitatively detects multiple IGF-1R family proteins, undoubtedly represents a powerful tool for drug and biomarker discovery.

## Materials and Methods

### Materials

All antibodies were purchased from either BD PharMingen (San Diego, CA, USA) or R&D Systems (Minneapolis, MN, USA) or prepared by RayBiotech (Norcross, GA, USA). IGF family proteins were from R&D or prepared by RayBiotech. Horseradish peroxidase-conjugated streptavidin was purchased from BD PharMingen. Glass slides were purchased from Corning (Corning, NY, USA).

### Preparation of samples

The tumor samples and matching paratumorous tissue samples (adjacent to tumor, 3–5 centimeter distance) were collected from 25 patients diagnosed with hepatocellular carcinomas (HCC) at the Department of Surgery, the First Affiliated Hospital, Sun Yat-Sen University, China. All procedures were consistent with the National Institutes of Health Guide and approved by institutional review board and Hospital Ethical Committee with patients' consent. Information about HCC diagnosis, staging, histology, grade and age was available, but the identity of patients (name, address, date of birth) was not provided. All samples were snap-frozen and stored at −80°C. Immediately after removal from freezer, the HCC and adjacent tissues were cut into smaller pieces on dry ice, then soaked in lysis buffer (10 mM pH 7.5, Tris.HCl, 25 mM NaCl, 1% sodium deoxycholate, 1% Triton X-100, containing phosphatase and protease inhibitors). The tissue samples were then pulverized using a handheld homogenizer (Power Gen 125, Fisher Scientific). Tissue lysates were centrifuged at 10,000 rpm for 10 minutes, and the supernatants were collected and stored at −80°C. Protein concentrations were determined using the bichinchonic acid (BCA) assay.

### IGF signaling antibody array technology

Capture antibodies were printed onto glass slide at a volume of 350 pL per spot at a pitch of 500 µm using a PerkinElmer (Shelton, CT, USA) non-contact Piezorray Arrayer. Four replicate spots were printed for each antibody; each slide contained 16 individual microarrays. Biotinylated anti-bovine IgG (goat) antibody was also printed as a detection control. The glass slides were fitted with 16-well gasketed hybridization chambers to prevent cross-contamination of microarrays. After drying at room temperature for 1–2 hours, the slides were blocked with 5% bovine serum albumin/PBS for 30 minutes. Individual arrays were then incubated with conditioned medium, human serum, tissues lysates or standard protein mixtures at varying concentrations (diluted in blocking buffer) overnight at 4°C. Slides were washed 3 times with wash buffer I (PBS, 0.1% Tween 20; 5 minutes per wash), and 2 times with wash buffer II (PBS; 5 minutes per wash) to remove unbound proteins. Slides were then incubated for 2 hours with a cocktail of corresponding antibody pairs (biotin-conjugated). Glass slides were washed and incubated with Cy3 equivalent dye-conjugated streptavidin (AnaSpec, Freemont, CA, USA) for 1 hour at room temperature, then washed as described above. After drying, the slides were scanned with a laser scanner (Genepix 4000B, Axon Instruments, Sunnyvale, CA, USA) and the signals were visualized.

### Western blot analysis

Western blotting was performed according to our previous publication [38]. Tissue lysates containing equal amounts of protein were analyzed by SDS-PAGE and proteins were transferred to polyvinylidene difluoride (PVDF) membranes (Millipore Corp., Bedford, MA). The membranes were then probed with monoclonal antibodies against IFGBP-2 and Beta Actin. Signals were detected with ECL system (Amersham Corp., Amersham, United Kingdom).

### Data Analysis

The signal densities of all spots from the arrays were extracted using Genepix Pro 6.1 software. Standard curves and expression levels of each IGF-IR family protein were calculated using Microsoft exel-based analysis tool developed by us. The array data of all tissue samples were normalized based on the positive control signals. The background threshold value was defined as mean signal intensity plus twice the standard deviation (SD) of 10 controls, where the arrays were assayed without any serum sample or standard protein. If the sample's signal intensity for a particular analyte was less than the background threshold, that analyte was removed from further analysis.

### Statistical Analysis

To test the significance of the protein expression differences between tumor samples and matching paratumorous tissue samples, adjusted student's t tests were applied using SSPS statistical software (SPSS, Inc., Chicago, IL, USA). For inclusion in further classification studies, the cut-off for statistical significance for each analyte was *P*<0.05. Normal distribution analysis was performed with both Kolmogorov-Sminov analysis and Shapiro-Wilk analysis (SSPS statistical software, SPSS, Inc., Chicago, IL, USA). Normal distribution was counted as *P*>0.05. Highly correlated cytokine markers of antibody array were selected for hierarchical cluster analysis.
